# Triterpenes as Potentially Cytotoxic Compounds

**DOI:** 10.3390/molecules20011610

**Published:** 2015-01-19

**Authors:** Malwina Chudzik, Ilona Korzonek-Szlacheta, Wojciech Król

**Affiliations:** 1 Chair and Department of Microbiology and Immunology, Medical University of Silesia in Katowice, Jordana 19, Zabrze 41-808, Poland; E-Mail: malwina.m.jarosz@gmail.com; 2 Department of Nutrition-Associated Disease Prevention, Faculty of Public Health, Medical University of Silesia in Katowice, Piekarska 18, Bytom 41-902, Poland; E-Mail: ikorzonek@sum.edu.pl

**Keywords:** triterpenes, cytotoxicity

## Abstract

Triterpenes are compounds of natural origin, which have numerously biological activities: anti-cancer properties, anti-inflammatory, anti-oxidative, anti-viral, anti-bacterial and anti-fungal. These substances can be isolated from plants, animals or fungi. Nowadays, when neoplasms are main cause of death, triterpenes can become an alternative method for treating cancer because of their cytotoxic properties and chemopreventive activities.

## 1. Introduction

Neoplasms are the main cause of death worldwide. Each year tumors are diagnosed in about 11 million people, ending with death in 7.6 million; the number forecasted for 2030 reaches 13.1 million. The major ways of cancer treatment are chemotherapy and radiotherapy, which unfortunately proved toxic to other living cells of the body [1]. Therefore, numerous studies have focused on application of natural products to prevent and to treat cancer. Among bioactive compounds, an important group is that of triterpenes, which show cytotoxic properties against tumor cells at low activity toward normal cells [2].

## 2. Origin, Classification, Chemical Structure and Role of Triterpenes

Triterpenes are naturally occurring alkenes of vegetable [3,4], animal [5,6] and also fungal [7,8] origin, classified among an extensive and structurally diverse group of natural substances, referred to as triterpenoids. Their structure includes 30 elements of carbon and they are constituted by isoprene units [9]. Taking into consideration the structure, triterpenes may be divided into linear ones—mainly derivatives of squalene, tetracyclic and pentacyclic, containing respectively four and five cycles, as well as two- and tricyclic ones [10]. Representatives of those show anti-cancer properties [11,12] as well as anti-inflammatory [13], anti-oxidative [14], anti-viral [15,16], anti-bacterial [17] and anti-fungal ones [18]. A good example could be the betulinic acid and its derivatives which have been investigated for their strong cytotoxic properties [19,20]. Other important representatives are the compounds originating from squalene, dammarane, lanostane, oleane (e.g., oleanolic acid), lupane (e.g., lupeol), ursane (e.g., ursolic acid) or triterpenoid sapogenins, for example cycloartane, friedelane, filicane and cucurbitane triterpenoids [21]. Table 1 gives examples of neoplastic cell lines sensitive to cytotoxic properties of triterpenes.

**Table 1:** Examples of neoplastic cell lines sensitive to cytotoxic properties of triterpenes.

Triterpene	Type of Neoplasm	Cytotoxicity Evaluation Method
Squalene derivatives	leukemia, melanoma, sarcoma, lung cancer, kidney cancer, cancer of the peripheral nervous system, colon cancer, breast cancer, ovarian carcinoma, cervical carcinoma, prostate cancer	MTT test, evaluation of apoptosis
Dammarane derivatives	glioma, lung cancer, ovarian carcinoma, colorectal carcinoma, colon cancer	MTT test, evaluation of apoptosis
Lanostane and its derivatives	leukemia, melanoma, glioma, gastric carcinoma, pancreatic cancer, colon cancer, hepatic cancer, lung cancer, breast cancer, ovarian carcinoma	MTT test, SRB evaluation of apoptosis
Lupeol	colorectal cancer, gastric cancer	MTT test, LDH evaluation of apoptosis
Oleanolic acid and its derivatives	thyroid carcinoma, ovarian carcinoma, breast cancer, colorectal cancer, glioma, leukemia, gastric adenocarcinoma	MTT test, evaluation of apoptosis
Betulinic acid and its derivatives	lung cancer, prostatic carcinoma, breast cancer, prostate cancer, ovarian carcinoma, cervical carcinoma, lung cancer, colorectal cancer, colon cancer, glioma, melanoma, thyroid tumor, colon adenocarcinoma, leukemia	MTT test, SRB evaluation of apoptosis
Ursolic acid and its derivatives	ovarian carcinoma, pancreatic carcinoma, prostate cancer, cervical carcinoma, hepatic cancer, breast cancer, colorectal cancer, leukemia, neuroma, colon adenocarcinoma	MTT test, SRB evaluation of apoptosis
Vegetal extracts	leukemia, melanoma, glioma, laryngeal cancer, breast cancer, hepatic cancer, gastric cancer, lung cancer, ovarian carcinoma, prostate cancer, colon cancer, epithelial carcinoma	MTT, evaluation of apoptosis
Fungal extracts	melanoma, lymphoma, glioma, breast cancer, ovarian carcinoma, prostate cancer, breast cancer, hepatic cancer, gastric cancer, colon cancer, epidermal nasopharyngeal carcinoma	MTT

## 3. Cytotoxicity and Evaluation Methods

Cytotoxicity defined toxicity of particular substances toward different cells. Evaluation of biological activity of compounds employs cellular models where evaluation bases upon the cellular line culture along with the tested substance and next, measurement of parameters associated with cell proliferation, e.g., quantity, ability to divide, mitochondrial activity, condition of the cell membrane or total content of DNA or proteins. Several stains are used to mark the cytotoxic activity: SRB (evaluation of total protein content as the stain binds with proteins), MTT (evaluation of oxydoreductive activity of mitochondria—measurement of ability to reduce the stain in living cells), DAPI (evaluation of total DNA content, using fluorochromes capable of stoichiometric binding with DNA), propidium iodide, erythrosin B and trypan blue (staining of dead cells). The use of fluorescent stains makes it possible to apply flow cytometry (measurement of intensity of light scattered by the labeled cells) as well as fluorescence microscopy (observation and counting of the labeled cells), thanks to which, it is possible to mark all: dead, living and apoptotic cells [22,23,24].

Measurement of triterpene cytotoxicity employs mainly MTT tests [25], SRB [26] and LDH (colorimetric assay of lactate dehydrogenase, expressed upon the cell membrane damage [27]) as well as evaluation of apoptosis and necrosis with fluorescence methods [28].

## 4. Cytotoxic Activity of Triterpenes

Organic compounds of natural origin, such as triterpenes, are substances produced by the tissues of higher plants, fungi, marine organisms and animals. They are characterized by high diversity of chemical structure and biological properties [11]. Table 2 presents chemical structure of major cytotoxic representatives of triterpenes.

**Table 2:** Examples of triterpenes presenting cytotoxic activity.

Triterpene	Chemical Structure	Triterpene	Chemical Structure
Squalene	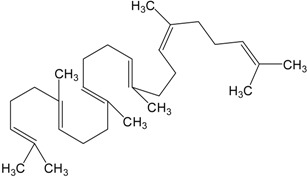	Dammarane	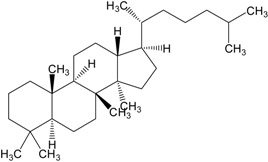
Ginsenoide	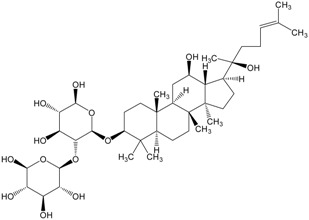	Lupane	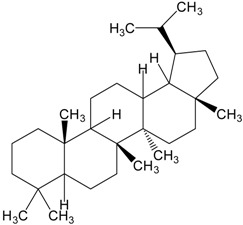
Lanostane	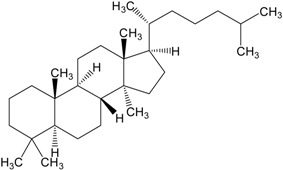	Lupeol	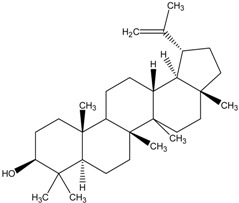
Oleane	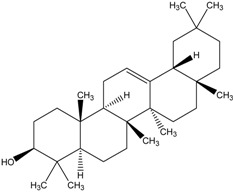	Oleanolic acid	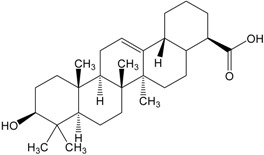
Betulinic acid	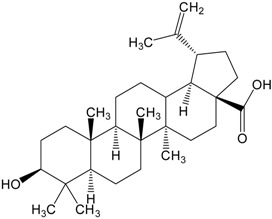	Ursane	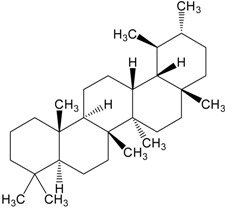
Ursolic acid	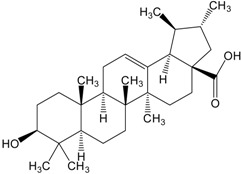	Isopropyl 3β-hydroxyurs-12-en-28-oat (ursolic acid derivative)	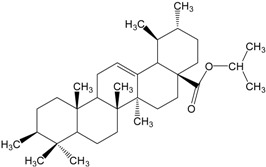
Cycloart-23E-ene-3β,25-diol (cycloartane troterpenoid)	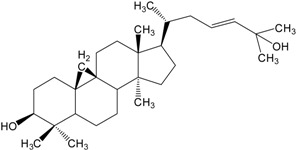	3-oxo-16β,29-hydroxy-firedelane (friedelane-type triterpene)	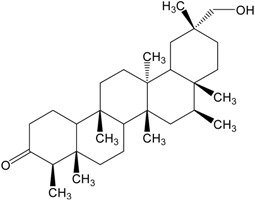
7α-hydroksyfern-8-en-11-one (fernane-type triterpenoid)	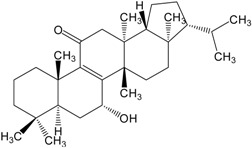	3β-hydroxyfilic-4(23)-ene (filicane-type triterpenoid)	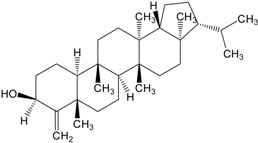
Hemslepenside A (cucurbitane troterpenoid)		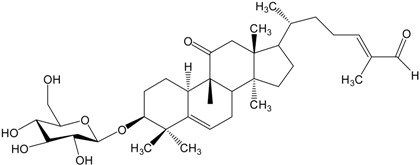	

Extracts of vegetal and fungal origin, containing triterpenes in their structures, have been studied for many years now, as potentially cytotoxic toward neoplastic cells. MTT tests have frequently shown cytotoxicity toward cell lines. Components of the extract from leaves and branches of *Juglans sinensis*, containing pentacyclic terpenes, showed cytotoxicity against line B16F10 (mice melanoma), Hep-2 (human larynx carcinoma), MCF-7 (breast cancer) and U87-MG (glioma) [29]. Triterpenes from *Clematis argentilucida* proved active against leukemic cells (HL-60), hepatic cancer (Hep-G2) and glioma (U251MG) [30]. Pentacyclic triterpenes isolated from *Liquidambar formosana* manifested strong cytotoxic properties against line MDA-MB435S (breast cancer) [31]. Compounds originating from *Dysoxylum cumingianum* showed cytotoxicity against cellular lines KB (epidermal nasopharyngeal cancer), MC7 (breast cancer) as well as multidrug resistant line KB-C2 [32]. A study on plants extracts is still ongoing because there are many triterpenes, which have not been described before. *Euphorbia macrostegia* extract contains four cycloartane triterpenoids which possess cytotoxic activity against two human breast cancer lines: MDA-MB48 and MCF-7 [33]. Branches of *Maytenus robusta* contains seven pentacyclic triterpenes (friedelane-type) presenting cytotoxicity against 4T1 cells (murine breast cancer cells) [34]. Moreover roots of *Hemsleya penxianesis* contains six cucurbitane triterpenoids with cytotoxic activities against human lung adenocarcinoma cells (H460) and colon cancer cells (SW-620, COLO205) [35]. Cytotoxicity is also tested with the use of other methods, e.g., SRB, as in case of *Pleurospermum kamtschaticum*. The isolated compounds showed activity against lung cancer cells (A549), ovarian cancer (SK-OV-3), cutaneous carcinoma (SK-MEL-2) and colon cancer (HCT15) [36]. Unfortunately, not all triterpenes isolated from plants, show cytotoxic properties (e.g., those from *Xanthoceras sorbifolia*) [37], while other are toxic to both, tumor cells and normal cells of the body (e.g., from *Maytenus undata*) [18]. Similar cytotoxic activity was observed in compounds from *Albizia inundata*, which proved toxic to both, melanoma cells (B16F10, KMEL28) and squamous cells of the neck and head (JAMAR, MDA1986) [38]. Tests comprised also triterpenes from *Euphorbia hirta*; two of them showed no cytotoxicity against the evaluated cell lines (HCT116 and A549), while one was toxic to both, tumor cells and normal ovarian cells of a hamster [8]. *Cimicifuga foetida* rhizomes contain many biologically active constituents, mainly triterpenoids and some of them have cytotoxic activity against human tumor cells lines: HepG2, SMMC-772 (hepatocellular carcinoma), HL-60 (leukemia), A549 (epithelial carcinoma), MCF-7 (breast cancer), SW-480 (colon carcinoma) and K 562 (myelogenous leukemia) [39]. Cytotoxic activity against HepG2 have also triterpenoids deriving from *Anthrodia cinnamomea* extract. This biological active compounds can induce apoptosis as well [40]. Extract from *Hibiscus syriacus* also contains triterpenoids with cytotoxic properties such as against lung cancer cells (A549) [41]. Triterpenoids from *Abies delavayi* show cytotoxic activity against A549 cell line as well and additionally against LoVo and Colo-205 (colorectal adenocarcinoma) [42]. Roots and leaves of *Ekebergia capensis* contain pentacyclic triterpenoids which are cytotoxic against human larynx carcinoma (HEp2), breast cancer (MDA-MB-231) and unfortunately “normal” (vero) cells [43]. From *Angiopteris palmiformis* isolated five triterpenoids (fernane- and filicane-type) and four of them were cytotoxic against cancer cell line SK Hep-1 (human heatoma) [44]. Very important phytochemicals are triterpenoid saponins, e.g., extract from *Bupleurum falcatum* contains cytotoxic compounds against breast cancer (MCF-7) and liver cancer (HepG2) [45]. Similar activity, against HepG2 and MCF-7 cell lines and also colon cancer cells (HCT116), show triterpenoids saponins from *Gleotitsia caspica* [46]. Cytotoxicity of the *Polycarpaea corymbosa* and *Gypsophila paniculata* extracts were measured by XTT assay. The triterpenoid saponins from these extracts were cytotoxic against SW480 (colorectal adenocarcinoma), DU145 (prostate cancer) [47] and human leukemia and lymphoma cell lines (HSB-2, Ramon and Daudi) [48] respectively. Not all triterpenoid saponins are cytotoxic against cancer cell lines. It depends on the chemical structure. Only few compounds from *Clematis argentilucida* are active against glioblastoma (U251MG) [49] as well as phytochemicals from *Patrinia scabra* roots against hepatic cancer (Hep-G2) [50]. Another important group of plant origin compounds are limonoids, which are highly oxygenated triterpenes classed as tetranotriterpenoids [51]. These biological active chemicals can be found in the *Walsura yunnanensis* leaves and twigs or *Melia azedarach* fruits. In the first case two of nine limonoids have cytotoxic properties against HL-60, SMMC-7721 (hepatocarcinoma), A549, MCF-7, SW480 [52], and in the second phytochemicals are active against HL60, A549, SK-BR-3 cell lines and induce apoptosis of AZ521 cells (stomach cancer) as well [53].

Apart from vegetal extracts, cytotoxic activity is shown also by triterpenes of fungal origin. Three compounds, isolated from *Daldinia concentrica*, were cytotoxic to cell lines KB, MCF7, SK-LU-1 (lung cancer) and HepG2 [54]. Similar activity was observed in six constituents from *Antrodia camphorata*. Cytotoxicity was evaluated using MTT test in lines: HSC-3 (oral squamous cell carcinoma), B16F1 and B16F10 (mice melanoma), Huh-7 (hepatic cancer), SKOV3 (ovarian carcinoma), MCF-7 (breast cancer), A-2058 (melanoma) [55].

Looking for potential sources of cytotoxic triterpenes, the scientists have recently turned to marine animals, such as sea cucumbers and sponges. A triterpenoid isolated from *Penares* sp., showed cytotoxic properties against leukemic cells (HL-60) [6].

One of the most important representatives of triterpenes is the betulinic acid. It has been proved many times to show selective, cytotoxic properties against tumor cells, with no activity toward normal cells of the body. MTT tests have shown cytotoxicity of the betulinic acid toward the following cell lines: Me665/2/21 and Me665/2/60 (melanoma), A2780, OVCAR-5 and IGROV-1 (ovarian carcinoma), A431 (cervical cancer), H4360 (non microcellular lung cancer), POGB (lung cancer) and POGB/DX (line resistant to doxorubicin). Activity towards cutaneous fibroblasts and peripheral blood lymphoblasts have also been evaluated. Toxicity of the betulinic acid toward those lined was 2–5 fold weaker than against the neoplastic lines [2]. Apart from the betulinic acid itself, similar, or even stronger, cytotoxic properties are shown by its derivatives, still manifesting no such properties toward normal cells of the body. Betulinic acid derivatives (and those of ursolic acid also) showed no cytotoxic activity toward human, embryonic kidney cells (HEK293T), which was proved by flow cytometry [56]. The betulinic acid and its derivatives (e.g., betulinic acid saponins) are considered as potential, clinical, anti-cancer agent, have been described for their selective cytotoxic activity. The compounds have proved active toward lung cancer (A549), colorectal carcinoma (DLD-1), breast cancer (MCF7) and prostate (PC-3) [57], as well as melanoma (518A2), anaplastic thyroid tumor (850c), ovarian carcinoma (A2780), lung cancer (A549) and breast cancer (MCF-7) [26,58], showing no cytotoxic activity toward cutaneous fibroblasts (WS1) [57] and colon fibroblasts [26]. New derivatives of the betulinic acid are still being defined. Their cytotoxic activity has also been evaluated for cell lines CEM (leukemia), CEM-DNR (line resistant to daunorubicin), K562 (leukemia), K562-TAX (line resistant to paclitaxel), HCT116p53-/- (line void of gene p53), BJ (cutaneous fibroblasts), MCR-5 (lung fibroblasts) [25] as well as SW707 (colorectal adenocarcinoma), CCRF/CEM (leukemia), T47D (breast cancer), P388 (mice leukemia) and BALB3T3 (mice fibroblasts) [20], as well as A-375 (melanoma), A431 (epidermoid carcinoma), SH-SYSY (neuroma), HT-29 (colon cancer), HepG2 (hepatic carcinoma), HeLa (cervical cancer), MIA PaCa-2 (pancreatic carcinoma) and Jurkat (leukemia) [27,59]. As earlier, the results have been promising, with high activity of the obtained derivatives at high selectivity toward neoplastic cells. Cytotoxicity of the above components was evaluated by MTT, SRB and XTT (calorimetric tests, similar to MTT) and apoptosis was also assessed.

The betulinic acid originates from lupane. This is a group of pentacyclic triterpenes, characterized by cytotoxic properties, which may be isolated from plants (e.g., *Spirostachys africana*) [60] or synthetized. Their activity has been proved for cell lines of lung cancer (A549), colorectal carcinoma (DLD-1), breast cancer (MCF-7) and prostate cancer (PC-3), at no activity toward cutaneous fibroblasts (WS1-1) [61]. Another representative of this group is lupeol. Cytotoxicity of lupeol has been evaluated by MTT tests along with the neoplastic cell apoptosis assay. The triterpene showed selective cytotoxic properties toward neoplastic lines, through inhibition of cellular life and growing number of apoptotic cells. Sensitivity was proved in case of hepatic cell carcinoma (HepG2 and SMMC7721) [62,63] and colorectal carcinoma (DLDS1, HTC116 and RKO) [64], while hepatic cells proved insensitive (Lo-9) [63]. Lupeol, not only inhibits life of neoplastic cells, but also stimulates NK cells, which together gave promising results pointing to an alternative anti-cancer drug. Such activity has been tested toward gastric carcinoma (N87, BGC823 and HGC27), with the use of MTT, LDH and flow cytometry [65].

Other representatives of pentacyclic triterpenes include ursolic and oleanolic acids. Using MTT tests, cytotoxic activity of the ursolic acid has been shown toward ovarian carcinoma (SKOV-3 and A2780) [66], pancreatic cancer (MIA PaCa-2, PANC-1 and Capac-1) [67] as well as prostate cancer (PC-3, DU145 m LNCaP) [68]. Studies comprised also derivatives of this triterpene. Unfortunately, not all derivatives show such cytotoxic properties, while other prove remarkably more active than the ursolic acid, e.g., toward leukemic cells (HL-60) [69], cervical carcinoma (HeLa), ovarian carcinoma (OVACAR-3), hepatic cancer (HepG2), gastric adenocarcinoma (BGC-823) or neuroma (SH-SYSY) [70]. Cytotoxicity of the ursolic acid derivatives has also been evaluated for the lines: MGC-803 (gastric carcinoma), Bcap-37 (breast cancer), NIH3T3 (mice fibroblasts) [71], as well as AGS (gastric carcinoma), HT-29 (colorectal cancer), IPC-3 (prostate cancer) [72]. Apart from MTT test, the effect of the evaluated compounds on apoptosis of neoplastic cells was assessed with the use of fluorescent microscopy and flow cytometry, which brought some encouraging results. Some of ursolic acid derivatives isolated from leaves of *Eriobotrya* *japonica* exhibit cytotoxic activity against human bladder cancer cell line (NTUB1), prostate cancer cell line (PC3) and epithelial carcinoma cell line (A549) [73]. Furthermore one of them (isopropyl 3β-hudroxyurs-12-en-28-oat) caused the death of NTUB1 cells via inducing apoptosis and showed significant antiproliferative activity against gastric cancer cells (BCG823) [74]. Just as in case of the betulinic acid, some of the derivatives proved more active than the original agent and showed high selectivity. The assays have been kept up-to-date, e.g., cytotoxicity of the ursolic and oleanolic acid was evaluated toward melanoma cells (518A2), thyroid cancer (8505C), lung cancer (A549), ovarian carcinoma (A2780), colorectal cancer (HT-29), breast cancer (MCF-7), WW030272 and the fibroblasts (NIH353). Activity of the triterpenes was evaluated with the use of MTT and SRB tests, showing that easy modifications may transform the compounds into agents manifesting some promising anti-cancer activity [75]. The oleanolic acid is also capable of inducing apoptosis in line HaCaT (immortalized keratinocyte cell line) at relatively low cytotoxicity, which points to potential anti-cancer properties of this compound [76]. As in case of other pentacyclic triterpenes, synthetized and looked for are derivatives of the oleanolic acid, supposed to show stronger cytotoxicity and selectivity as well as better capabilities of inducing apoptosis. Such properties were evaluated for lines PC-3 (prostate cancer), MCF-7 (breast cancer), A549 (lung cancer), BGC-823 [77], HL-60 (leukemia), A375 (renal carcinoma) [78], HepG2 (gastric carcinoma) [79]. Some of the derivatives showed properties slightly worse than the oleanolic acid, some, on the other hand proved much better, which pointed to the most effective modifications. Cytotoxicity has also been evaluated in triterpenes from oleane-type and isolated from plants. Compounds from *Fatsia polycarpa* were tested for HepG2 to show cytotoxic properties depending on a given triterpene [80]. Another evaluated plant was *Anemone taipaiensis*, which showed cytotoxic activity of the oleane-type triterpenoids toward cell lines HepG2, HL-60, A549, HeLa (cervical carcinoma), and U87-MG (glioma) [81]. Cytotoxic properties possess also oleane-type saponins e.g., originating from *Anemone rivularis* rhizomes. All extract’s compounds are active against leukemia (HL-60), hepatocellular carcinoma (HepG2) and lung cancer (A549) [82].

Cytotoxic properties are also shown by tetracyclic triterpenes, Tetracyclic terpenes from *Ailanthus altissima* inhibited life od gastric carcinoma cells (BCG-823, KE-97), nasopharyngeal carcinoma (KB), liver cancer (Huh-7) and lymphoma (Jurkat) [83], while derivatives of ginsenoside (compounds originating from dammarane), not only inhibited growth of colorectal cancer cells (HCT116, HT29, LoVo and SW480) but also induced apoptosis [84]. An important group of tetracyclic compounds is constituted by triterpenes originating from lanostane. Lanostane, as well as some of its derivatives, showed very poor activity toward neoplastic cell lines U87 (glioma) [85], A549 (lung cancer), DLD-1 (colorectal carcinoma) and lack of cytotoxicity toward normal cutaneous cells (WS1) [86]. On the other hand, many lanostane –type triterpenes have been isolated from fungi, showing selective growth inhibitory properties as well as induction of apoptosis of neoplastic cells. An example is *Poria Cocos*, source of compounds cytotoxic toward lines DU145 (prostate cancer) and A549 (lung cancer) [87], as well as HL-60 (leukemia), NIH:OVAE-3 (ovarian carcinoma), SK-BR-3 (breast cancer), CRL1579 (melanoma), A2521 (gastric carcinoma) and PANC-1 (pancreatic carcinoma) [88]. Another good example is *Tilia kiusiana* which contains lanostane-type triterpenoids cytotoxic against HeLa and HL-60 cell lines [89]. Tetracyclic compounds from fungi have proved cytotoxicity toward many neoplastic cells; those from *Naemataloma fasciculare* are active toward lung cancer cells (A549), ovarian carcinoma (SK-OV-3), melanoma (SK-MEL-2) and colon cancer (HCT-15) [7]. *Antrodia Camphorata* is the source of tetracyclic triterpenes, showing selective properties, as they inhibit growth of neoplastic cells and induce apoptosis, while normal cells are not sensitive to them. Such properties are different, depending on the compound or the cell line, the relations however, remain the same. Sensitivity has been observed in neoplastic lines of colon cancer (HT-29, HCT-116, SW480), liver cancer (Huh7, HepG2, Hep3B), breast cancer (MDAMB231, MCF-7) as well as lung cancer (A549, CL1-0), while lines MCF10A (mammary epithelial cells) and HS68 (prepuce fibroblasts) remain insensitive to the effect of such triterpenes [90].

Among other sources, the plants deliver dammarane-type tetracyclic triterpenes. The studies performed have pointed to their potential role in prevention and treatment of cancer. *Bacopa monniera* brought compounds inhibiting life of cell lines MDA-MB-231 (breast cancer), SGH-44 (glioma), A549 (breast cancer), HCT-8 (adenocarcinoma) and PC-3M (prostate cancer) [91]; triterpenes from *Panax ginseng* proved cytotoxic against breast cancer cells (MCF-7), prostate cancer (DU145), liver cancer (HepG2), colon cancer (Colon205), lung cancer (A549) and leukemia (HL-60) [92], while those from *Gynostemma pentaphyllum* were active toward lines HL-60, A549, MCF-7, HT-29 (colon cancer) and SK-OV-3 (ovarian carcinoma) [93]. Apart from inhibition of the neoplastic lines, ginsenoids induce apoptosis of neoplastic cells (HL-60) [94]. Unfortunately, not all representatives of such compounds show cytotoxic properties. For example, compounds from *Combretum inflatum* proved no activity against lung cancer cells (NCI-H460) [95], and those from *Panax quinquefolium* were ineffective toward breast cancer cells (MCF-7) [96].

Cytotoxic activity has also been observed in derivatives of linear squalene. Triterpenes isolated from algae showed cytotoxic activity toward leukemia (Jurkat), sarcoma (CADO-ES-1), cervical carcinoma (HeLa) and multiple carcinoma (MM1440) [97]. Apart from the inhibitory role, squalene compounds show also the capability of inducing apoptosis in many neoplastic lines: leukemia, melanoma, colon cancer, prostate cancer, ovarian carcinoma, liver cancer, breast cancer, lung cancer and peripheral nervous system carcinoma [98], therefore, may be investigated as potential, alternative agents in cancer treatment.

## 5. Conclusions

Triterpenes are natural compounds showing a wide spectrum of biological effects. They proved to have anti-bacterial, anti-viral, anti-fungal, anti-oxidative and anti-inflammatory properties, as well as anti-cancer and chemopreventive ones. Not only they are capable of inhibiting life of neoplastic cell lines, but also induce apoptosis of cancer cells, to cause their “suicidal” death, with no threat to normal cells of the body. Such properties, in particular the selectivity of triterpenes’ activity, present them as alternatives in cancer treatment and prevention. Therefore, it is essential to gain such compounds, for evaluation of their cytotoxic properties and underlying mechanisms, through synthesis of new derivatives.
